# The Association of Paraoxonase-1 Polymorphism with Carotid Artery Stenosis among Elderly Chinese Population

**DOI:** 10.1155/2020/3084120

**Published:** 2020-02-15

**Authors:** Jianyun Sun, Long Wang, Qian Yang, Taofeng Zhou, Xianhui Ding, Ke Yang, Zhiming Zhou

**Affiliations:** ^1^Department of Neurology, Yijishan Hospital, The First Affiliated Hospital of Wannan Medical College, Wuhu 241000, China; ^2^Department of Cardiology, The No. 2 People's Hospital of Hefei, Hefei 230000, China; ^3^Department of Neurology, The First Affiliated Hospital of Wannan Medical College, Wuhu 241000, China

## Abstract

Elderly population is in high risk of carotid atherosclerosis and artery stenosis (CAS). It has been proved that PON1 polymorphism is associated with low-density lipoprotein (LDL) oxidation, which plays an important role in artery atherosclerosis. CAS is an important cause of ischemic stroke. This study is aimed at investigating the association of PON1 (rs662) polymorphism with the risk of CAS among elderly Chinese population. Consecutive elderly patients with CAS were enrolled into the study. Genotyping for PON1 (rs662) polymorphism was performed on all participants. There were 310 CAS patients in this study, with 88 symptomatic CAS and 222 asymptomatic CAS. G allele had a frequency of 59.66% in symptomatic CAS (sCAS); and A allele had an incidence of 36.93% in asymptomatic CAS (aCAS) (*P* < 0.05). In all CAS patients with and without symptom, no associations were found in any genotype comparison. However, among aCAS subjects, based on GA phenotype, the odds ratio (OR) of the mutant GG with stenosis severity was 0.20 (*P* = 0.01). The OR of GG+GA mutation was 0.28 for moderate/severe severity, compared with GA type (*P* = 0.03). This study indicates that PON1 (rs662) polymorphism is not associated with the presence of symptom among CAS patients. Moreover, PON1 (rs662) polymorphism correlates with stenosis severity among aCAS.

## 1. Introduction

Carotid artery stenosis (CAS) is a well-defined factor of stroke risk [1], which accounts for approximately 10-15% of population with ischemic stroke [2]. Elderly population is in high risk of artery atherosclerosis and subsequent stenosis pathology. Low-density lipoprotein (LDL) is the main carrier of cholesterol across the body. Elevated LDL has been recognized as the inducer of atherosclerosis [3]. The oxidized LDL plays an important role in the atherosclerosis change in the artery system by inducing inflammation and lipid deposition [4].

Human paraoxonase (PON) is primarily synthesized in the liver, and further, it is produced into circulation with associations with high-density lipoprotein (HDL) activity [5, 6]. PON1 gene is discovered on the chromosome 7 between q21.3 and q22.1 [7]. PON1 is a calcium-dependent esterase involved in the activity of HDL, which is capable of hydrolyzing a wide range of subtracts [8]. It combines with arylesterase and catalyses the hydrolysis of organophosphate esters, aromatic carboxylic acid esters, and carbamates [9]. PON1 is proved to be associated with the hydrolysis of oxidized phospholipids in oxidized LDL [10]. It inhibits the oxidation of LDL and prevents the subsequent atherosclerosis change in the carotid artery [11]. There are several polymorphisms in the PIN1 gene. The rs662 SNP leads to transition between adenine and guanine nucleobases that results in glutamine-to-arginine substitution at codon 192 (Q192R) [12]. The genotype AA is linked with an unfavorable lipid status and low PON1 activity in patients [13]. However, the associations of PON1 polymorphism with CAS risk remain unknown.

The aim of this study was to evaluate the correlation of PON1 polymorphism in CAS among elderly Chinese population.

## 2. Materials and Methods

### 2.1. Population

The study population consisted of elderly subjects (65-80 years old) admitted to our hospital during January 2016 to January 2019. All patients received ultrasound, computed tomography angiography (CTA), or digital subtraction angiography (DSA) examination on the carotid artery. Carotid artery stenosis was defined using the criteria previously published [14]. According to the current guidelines and studies, symptomatic CAS (sCAS) is defined as the association with symptoms in the preceding 6 months; and asymptomatic CAS (aCAS) is seen as no prior symptoms can be identified or when symptoms occurred >6 months ago [14, 15]. The informed written consent was obtained from all subjects upon enrollment. The study protocol was approved by the institutional ethics committee of Yijishan Hospital, Wannan Medical College. Demographic characteristics, medical history (smoking, alcohol use, hypertension, diabetes, and acute coronary disease), and the severity of CAS are recorded in Table 1.

### 2.2. PCR

The protocol was reported by our previous study [16]. Generally, the genotype of venous blood was determined through nonamplified fluorescence staining in situ hybridization (Beijing Precision Medical Platform analysis software package). Peripheral blood samples (3-6 ml) were collected in EDTA-contained tubes. The pretreatment solution (10 × NH_4_Cl) and double-distilled water (1 : 9) were added into the working fluid (1 × NH_4_Cl). Then, 1.2 ml of the working fluid was added to 1.5 ml centrifuge tubes, and subsequently, the blood sample (150-200 *μ*l) was moved into the tube. The supernatant was removed after centrifugation at 3000 rpm. Add 30-50 ml of nucleic acid purification regents (Beijing Huaxia Times Gene Technology Co., Ltd.) after the precipitate to get a leukocyte suspension. Then. 1.5 *μ*l of the suspension was added to a digoxin dyeing solution (Beijing Huaxia Times Gene Technology Co., Ltd.), mixed, and centrifuged for a short time. After that, the tube cap was sealed tightly, and the tube was placed into a fluorescent detector. The signal intensity was examined by fluorescence in situ hybridization and chromosome karyotype analysis system to obtain the fluorescence curve. The PON1 (rs662) gene locus was genotyped.

### 2.3. Statistical Analysis

Data were analyzed using SPSS 23.0 software. Variables were presented as mean ± standard deviation (SD), median with interquartile ranges (IQR), or frequencies. A *χ*^2^ was used to compare categorical variables. Student's *t*-test or MU test was applied for continuous variables. Multivariable regression analysis was done to assess the associations between PON1 polymorphism and CAS with and without symptom, as well as the relations of PON1 with CAS severity. All tests of significance were two-sided with a *P* value less than 0.05.

## 3. Results

### 3.1. The Baseline Characteristics of CAS Participants

**Figure 1:**
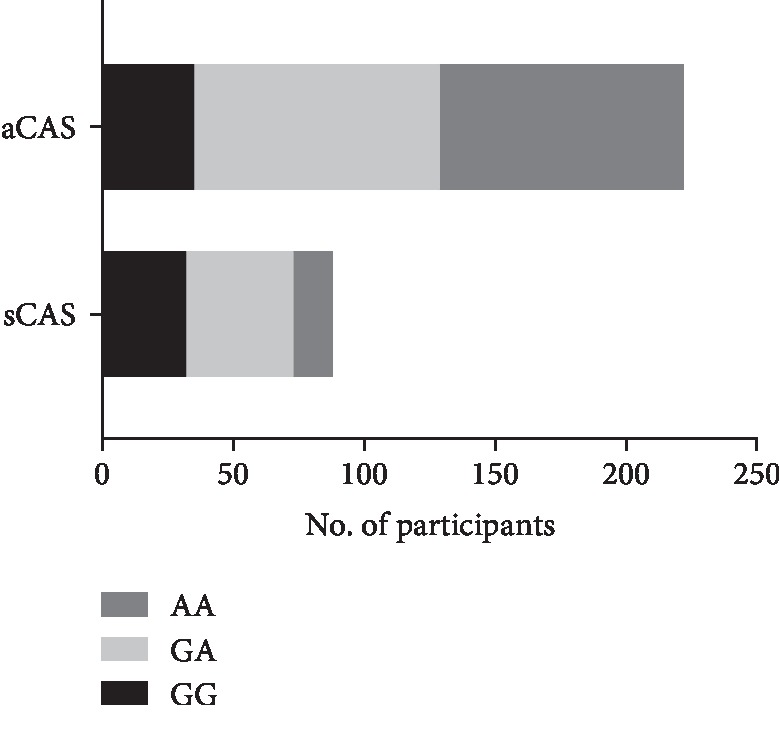
The genotype distribution of PON1 (rs662) polymorphism in all CAS patients. CAS: carotid artery stenosis.

**Figure 2:**
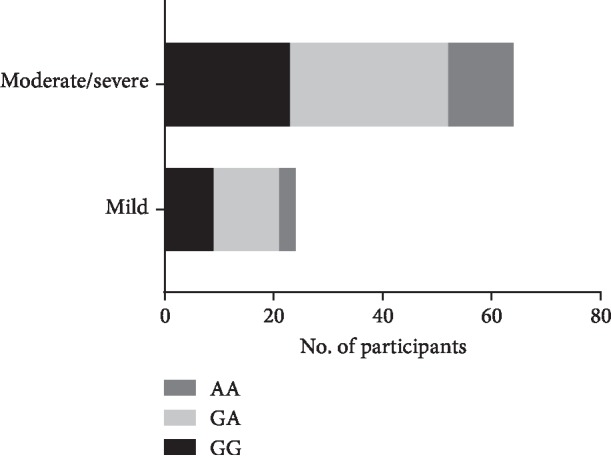
The genotype distribution of PON1 (rs662) polymorphism in sCAS patients. sCAS: symptomatic carotid artery stenosis.

**Figure 3:**
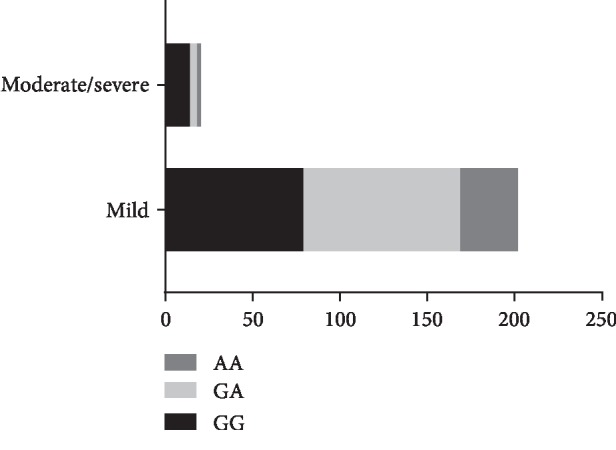
The genotype distribution of PON1 (rs662) polymorphism in aCAS patients. aCAS: asymptomatic carotid artery stenosis.

The baseline characteristics of the participants in this study are shown in Table 1. There were 345 patients in this study (204 males and 87 females). Among them, 92 patients were classified as symptomatic CAS and 253 subjects were in the asymptomatic CAS group. sCAS intended to have a higher rate of male ones (78.41% vs. 60.81%) and more smoking ones (22.73% vs. 11.71%), compared to aCAS (*P* < 0.05). The results of genotyping for PON1 in all CAS patients are shown in Figure 1. Moreover, there were 32 GG, 41 GA, and 15 AA mutation cases in sCAS (Figure 2); and 35 GG, 94 GA, and 93 AA mutations were found in aCAS (Figure 3). The distribution frequency of PON1 genotypes between them was not significant (*P* = 0.67). Moreover, G allele had a frequency of 62.87% in sCAS and 36.94% in aCAS (*P* < 0.05).

### 3.2. The Performances of PON1 Polymorphisms in Symptomatic and Asymptomatic CAS

We also showed the characteristics of sCAS and aCAS based on the severity of artery stenosis (Table 2). In sCAS, the frequencies of G were 62.50% and 58.59% in the mild and moderate/severe groups, respectively. Meanwhile, G had a frequency of 61.39% and 80.00% in the mild and moderate/severe groups, respectively, among aCAS population. For sCAS, the proportions of GG, GA, and AA in the mild severity group were 9 (37.50%), 12 (50.00%), and 3 (12.50%), whereas those in the moderate/severe subgroup were 23 (35.94%), 29 (45.31%), and 12 (18.75%), respectively. No differences were found in the overall comparison (*P* = 0.78). When we analyzed the proportions of GG, GA, and AA in mild aCAS, they were 79 (39.11%), 90 (44.55%), and 33 (16.34%); and those in the moderate/severe subgroup were 14 (70.00%), 4 (20.00%), and 2 (10.00%), respectively. There were significant differences among groups (*P* = 0.03).

### 3.3. Associations between PON1 Polymorphisms and CAS

Multivariate regression analysis was done to investigate the association between PON1 genotype and CAS (Table 3). In the overall analysis of CAS with and without symptom, no associations were found in any comparison. Also, no significances were discovered in all CAS sufferers, when analyzing the relations between CAS severity and polymorphic forms of PON1. However, we found that in aCAS, based on GA phenotype, the odds ratio (OR) of the mutant GG with stenosis severity was 0.20 (*P* = 0.01). And the OR of GG+GA mutation was 0.28 for moderate/severe severity, compared with GA type (*P* = 0.03).

## 4. Discussion

The current study analyzed the polymorphic types of PON1 (rs662) in CAS. It is well known that atherosclerosis is a common pathological change among elderly population [17]. Atherosclerosis and stenosis of the carotid artery pose a high risk of ischemic stroke among these elderly subjects [18]. CAS has been proved to be associated with dyslipidemia, vascular inflammation, and oxidative stress [19–21]. High level of LDL is a major cause for this pathology; the oxidation of LDL enhances the inflammatory status in the vascular system [22]. PON1 is an antioxidant enzyme bounded with HDL and could alleviate the oxidation of LDL [23].

It has been validated that the polymorphism of this gene in rs662 locus is associated with cardiovascular disease [24]. We found in the study that a higher rate of G presence existed in aCAS, compared with sCAS. However, the differences of G allele presence between CAS severity among sCAS and aCAS did not keep the same trend. We then speculated that the G/A allele was not associated with CAS severity. But the sample size was quite limited, which might hinder the credibility of the results.

Importantly, we investigated the associations of PON1 genotypes with CAS severity in those symptomatic and asymptomatic ones. We found that, compared to those with GG type, aCAS with GG type tended to have a low risk to bear moderate/severe severity (mild severity as reference: OR: 0.20 (0.06, 0.67)). Our findings indicated that PON1 polymorphism was associated with the severity of aCAS. This is consistent with previous understanding of the role of PON1. It has been proved that PON1 plays an important role in antioxidant activity among diabetic patients [7, 25]. PON1 regulates HDL-mediated cholesterol efflux from macrophages [26]. The rs662 SNP is the one that could induce the substitution of Q192R and contributes to lower PON1 activity [24]. Thus, it is believed that GG phenotype is associated with decreased oxidative stress, reducing the risk of atherosclerosis. However, no associations were found among sCAS patients. Previous studies have indicated that sCAS and aCAS have different plaque formation and characteristics. We then speculated that PON1 was associated with the features of plaques in CAS and other atherosclerosis diseases.

However, among the subgroup of aCAS, we only found that GG+AA type was associated with a low risk of moderate/severe severity (mild severity as reference: 0.28 (0.09, 0.85)). This is quite contrary to the current understanding of the antioxidant effects of GG and AA genotypes. So PON1 (rs662) might participate in the formation of CAS through other pathways.

We here address some limitations of the study. Only single-center participants were enrolled, with relatively small sample size. This might be improved by this ongoing study. Also, it is a cross-sectional study. It will be more convincing with the longitudinal analysis of stenosis severity. The comprehensiveness of the PON1 study could be better if other polymorphic types of PON1 could be studied.

## 5. Conclusion

In conclusion, this study indicates that PON1 (rs662) polymorphism is not associated with the presence of symptom among CAS patients. Moreover, PON1 (rs662) polymorphism correlates with stenosis severity among aCAS.

## Figures and Tables

**Table 1:** The baseline characteristics of the study participants.

Variables	sCAS	aCAS	*P*
No. of subjects	88	222	—
Age (years)	72.14 ± 4.65	71.68 ± 4.44	0.43
Gender (M)	69	135	0.002
Smoking	20	26	0.02
Alcohol	7	14	0.62
HTD	174	68	0.88
DM	30	53	0.09
ACS	20	35	0.19
PON1	—	—	0.67
GG	32	35	—
GA	41	94	—
AA	15	93	—
G/A	105/71	164/280	<0.001
CAS severity	—	—	<0.001
Mild	24	202	—
Moderate	22	12	—
Severe	42	8	—

sCAS: symptomatic carotid artery stenosis; aCAS: asymptomatic carotid artery stenosis; M: male; F: female; DM: diabetes mellitus; ACS: acute coronary syndrome.

**Table 2:** The comparative analysis of the symptomatic and asymptomatic CAS.

Variables	sCAS		*P*	aCAS		*P*
	Mild	Moderate/severe		Mild	Moderate/severe	
No.	24	64	—	202	20	—
Age (years)	71.71 ± 4.39	72.67 ± 4.50	0.036	72.12 ± 4.65	72.30 ± 4.75	0.87
Gender (M)	18	51	1.00	116	19	0.001
Smoking	7	13	0.40	20	6	0.02
Alcohol	3	4	0.39	10	4	0.03
HTD	15	53	0.05	157	17	0.58
DM	10	20	0.45	50	3	0.42
ACS	3	17	0.25	30	5	0.33
PON1	—	—	0.78	—	—	0.03
GG	9	23	—	79	14	—
GA	12	29	—	90	4	—
AA	3	12	—	33	2	—
G/A	30/18	75/53	0.73	248/156	32/8	0.03

sCAS: symptomatic carotid artery stenosis; aCAS: asymptomatic carotid artery stenosis; M: male; F: female; DM: diabetes mellitus; ACS: acute coronary syndrome.

**Table 3:** The association between PON1 (rs662) polymorphism and CAS severity.

	Symptomatic		CAS severity					
Comparisons	All CAS	*P*	All CAS	*P*	sCAS	*P*	aCAS	*P*
	OR		OR		OR		OR	
AA vs. GG	1.26 [0.72, 2.22]	0.42	0.48 [0.22, 1.04]	0.06	1.24 [0.40, 3.83]	0.71	3.08 [0.62, 15.19]	0.17
AA vs. GA	1.31 [0.62, 2.80]	0.48	0.70 [0.25, 1.91]	0.48	2.21 [0.42, 11.65]	0.35	0.61 [0.10, 3.72]	0.59
GA vs. GG	0.96 [0.46, 2.02]	0.92	0.68 [0.25, 1.86]	0.46	0.56 [0.12, 2.66]	0.47	0.20 [0.06, 0.67]	0.01^∗^
GG vs. GA+AA	1.28 [0.75, 2.16]	0.37	1.76 [0.85, 3.66]	0.13	0.50 [0.12, 2.12]	0.34	3.01 [0.37, 24.67]	0.30
GA vs. GG+AA	0.86 [0.51, 1.44]	0.55	0.64 [0.31, 1.31]	0.22	1.29 [0.46, 3.62]	0.63	0.28 [0.09, 0.85]	0.03^∗^
AA vs. GG+GA	0.86 [0.43, 1.72]	0.68	0.81 [0.31, 2.13]	0.66	1.14 [0.43, 3.01]	0.79	2.55 [0.77, 8.50]	0.13

## Data Availability

The data used to support the findings of this study are available from the corresponding author.
